# Pattern of Timing Adherence Could Guide Recommendations for Personalized Intake Schedules

**DOI:** 10.3390/jpm2040267

**Published:** 2012-11-28

**Authors:** Philipp Walter, Isabelle Arnet, Michel Romanens, Dimitrios A. Tsakiris, Kurt E. Hersberger

**Affiliations:** 1 Pharmaceutical Care Research Group, Department of Pharmaceutical Sciences, University of Basel, Pharmazentrum, Klingelbergstrasse 50, CH-4056 Basel, Switzerland; E-Mails: isabelle.arnet@unibas.ch (I.A.); kurt.hersberger@unibas.ch (K.E.H.); 2 Vascular Risk Foundation VARIFO, Ziegelfeldstrasse 1, CH-4600 Olten, Switzerland; E-Mail: info@kardiolab.ch; 3 Diagnostic Hematology, University Hospital of Basel, Petersgraben 4, CH-4031 Basel, Switzerland; E-Mail: TsakirisD@uhbs.ch

**Keywords:** compliance, adherence, time variability, electronic polymedication monitoring, lipid lowering agents

## Abstract

Deviations in execution from the prescribed drug intake schedules (timing non adherence) are frequent and may pose a substantial risk for therapeutic failure. Simple methods to monitor timing adherence with multiple drugs are missing. A new technology, *i.e.*, the polymedication electronic monitoring system (POEMS) attached to a multidrug punch card, was used in a clinical trial on outpatients with prescribed medicines for vascular risk reduction. The complete delineation of timing adherence allows for the calculation of objective adherence parameters and the linking of exposure with drug-drug interactions. A sub-analysis was performed on 68 patients, who were prescribed lipid lowering therapy. A smaller intake time variability of the lipid lowering drug was significantly associated with better levels of LDL-cholesterol, independently of the time of day. This finding may challenge current general recommendations for the timing of lipid lowering drugs’ intake and substantiate that inter-individual differences in timing adherence may contribute to response variability. Thus, objective parameters based on multidrug adherence monitoring should be considered as independent variables in personalized medicine. In clinical practice, personalized intake recommendations according to patients’ pattern of timing adherence may help to optimize the effectiveness of lipid lowering agents.

## 1. Introduction

Patient non-adherence and shortcomings in timing adherence with prescribed drug regimen poses a substantial risk for therapeutic failure, regardless of the disease or patient characteristics [1]. Non-adherence is the result of multiple factors that have been classified into five dimensions [2]. Therapy-related factors, such as co-medication, dosing frequency and intake schedules, are likely to affect the execution of the patients’ therapy plans. Numerous direct and indirect methods for adherence measurement have been described [3]. More than 20 years of research on electronic adherence monitoring revealed several patterns of adherence, however focusing only on single drugs [4,5]. Electronic adherence monitoring proved to be the most sensitive method for adherence assessment and provided the best predictor of health outcomes [6,7]. The recently introduced polymedication electronic adherence monitoring system (POEMS) allows for monitoring of the intake of all oral solid drugs [8]. The complete delineation of timing adherence with any of the prescribed oral solid drugs allows for assessing whether specific adherence parameters are associated with biomarker outcomes, which are predictive of effectiveness and toxicity. Taking non-adherence is often arbitrarily defined as 80% of doses taken, regardless of the drug, although the rationale for drug-specific and more sophisticated cut-offs could be deducted from pharmacokinetic and pharmacodynamic characteristics [9]. Continuous variables for timing adherence can be helpful to overcome this imprecision. Time variability of drug intake (t_VAR_) was introduced to describe intra-individual intake variation [10]. Except for oral contraceptives, little is known about the impacts of intake time deviation on drug effectiveness, and no advice can be retrieved from drug labels on what should be undertaken if time deviations or missed doses occur. Pharmacodynamic biomarkers as intermediate outcomes can help to study the tolerability of time deviations in the execution of the drug regimen [11]. Low density lipoprotein cholesterol (LDL-C) is a well-established biomarker that reflects the effectiveness of lipid lowering therapy with statins, and substantial gaps to LDL-C target achievement have been reported [12]. The impact of adherence patterns on LDL-C values was analyzed in the context of a prospective trial on antiplatelet resistance in which adherence was monitored with POEMS [13]. The results presented in this article describe the intake characteristics of an outpatient cohort, their association with the treatment schedule, subjective measures of adherence and biomarker response in lipid lowering therapy.

## 2. Methods

The parent trial on antiplatelet resistance (ClinicalTrials.gov ID: NCT01039480) was approved by the cantonal ethics committee of Aargau, Switzerland and included patients with a prescription for aspirin and/or clopidogrel, recruited by general practitioners. Patients with a full set of data were included in the analysis, and sub-analysis concerned users of a lipid lowering drug (LLD). Levels of LDL-cholesterol (LDL-C) were used as surrogate outcome for therapeutic effectiveness. All the patients’ oral solid drugs were repacked into a multidrug punch card (Pharmis GmbH, Beinwil am See, Switzerland) with 7 × 4 units-of-use for seven days. The backside was covered with a polymer film, which registered the drug removal from each unit-of-use. The POEMS technology consists of imprinted electronic components that measure the electrical resistance and record the time of its changes when a loop is broken, *i.e.*, when a cavity is emptied. The patients were advised to take their drugs at the time they were normally used to and to return the punch card upon their second visit after one week. Removal of drugs on demand was recorded, but not considered for analysis. Individual intake schemes were analyzed, regardless of the prescribed treatment schedules. The following parameters were derived from the electronic reports and calculated as follows:

- (a) Time variability of drug intake (t_VAR_) according to Equation (1) [10]. 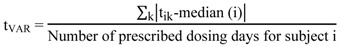
(1)
- (b) Dose-to-dose intervals as the time difference between two consecutive removals.
- (c) Weekend effects as the differences between objective adherence parameters on working days (Monday to Friday) and weekend days (Saturday and Sunday).

Patients’ subjective adherence scores were obtained with the Morisky-8 (MMAS-8, score 0 to 8) and the Beliefs about Medicines (BMQ) questionnaires [14,15]. Subscores for BMQ necessity (score 5 to 25), BMQ concerns (score 5 to 25) and BMQ differential (score −20 to +20) were calculated according to the authors [14]. In brief, higher scores are associated with better adherence.

Blood samples were analyzed with a Coulter® AcT_Diff_ (Beckman Coulter Inc., Brea, CA, USA) for hematology and Cobas® 6000 (Roche Diagnostics Inc., Rotkreuz, Switzerland) for clinical chemistry. Target LDL-C levels were set at 3.4mmol/L and 2.6mmol/L for primary and secondary prevention, respectively. The lipid lowering potency of the prescribed drugs were classified in five groups according to equivalence dose tables in order to control for uneven distribution in the statistical analysis [16].

### Statistical Analysis

Values are given as mean ± SD, median, quartiles and percentages where appropriate. Differences between patient groups were analyzed with unpaired t-Tests and the Mann-Whitney U-test, where applicable. Time variables were treated as scaled variables; objective adherence parameters were calculated and compared in a bivariate model using the Spearman rank correlation. A one way ANOVA, followed by post hoc LSD test, was used to compare differences of mean intake times between days. Two-tailed *p*-values ≤0.05 were considered significant.

## 3. Results

### 3.1. Patient Characteristics

The principal study, conducted between June 2010 and June 2011, was completed by 82 patients. Full sets of data were obtained for 78 patients. The study sample (30.8% women, mean age 66 ± 10 years) consisted of 44 patients (56.4%) with a history of arteriovascular events, and 34 patients (43.6%) were prescribed antiplatelet agents for primary prevention. Patients were prescribed one to 13 (median: five) drugs for oral intake, to take once a day (32 patients, 41.0%), twice (35 patients, 44.9%), thrice (eight patients, 10.3%) or more than thrice daily (three patients, 3.8%) (see Table 1 for more details). Higher dosing frequencies correlated strongly with a higher number of prescribed drugs (R^2^ = 0.61; *p* < 0.001). Antihypertensives were prescribed in 63 patients (82.9%), and 15 patients (19.2%) had an antidiabetic co-medication. Sixty-eight patients (87.2%) received LLD and attained mean LDL-C values of 2.3 ± 0.6 mmol/L (primary prevention; target values <3.4 mmol/L) and 2.5 ± 0.7 mmol/L (secondary prevention; target values <2.6 mmol/L).

**Table 1:** Therapy plan characteristics for n = 78 patients with full sets of data.

*Dosing frequency*	Number of drugs		Treatment schedule				N	%
	Median	Range	Morning	Midday	Evening	At night		
1 × daily	3.5	1–7	X				30	38.5
				X			1	1.3
						X	1	1.3
2 × daily	5.0	2–11	X		X		30	38.5
			X	X			2	2.6
			X			X	2	2.6
				X		X	1	1.3
3 × daily	7.0	3–10	X	X	X		5	6.4
			X	X		X	3	3.8
4 × daily	11.0	6–13	X	X	X	X	3	3.8

The median MMAS-8 score was 8.0 (range 4.5–8.0) and indicates a high adherence; the maximum score was reached by 53 patients (67.9%). BMQ subscores revealed a high perception of necessity (median 20; range 6–25) and little concerns (median 8; range 5–20). Patients with secondary prevention had moderately higher MMAS-8 scores (7.7 ± 0.6 *vs*. 7.3 ± 0.9; *p* = 0.06) and significantly higher BMQ necessity subscores (20.4 ± 4.0 *vs*. 17.9 ± 4.2; *p* = 0.01) than patients with primary prevention. The BMQ concerns score did not differ between these groups.

### 3.2. Objective Measures of Adherence

The prescriptions of the 78 patients theoretically involved 962 drug removals to be executed during the study participation. All dispensed punch cards were returned at the final visit (100% return rate). Visual inspection performed by the investigator confirmed that all removals were executed, but 47 events were not recorded (4.9% missing data), and 30 events could not be assigned to a drug removal even after a post hoc interview-based verification (3.1% implausible data) due to a deficiency in the recording technology. 

See Table 2 for the parameters describing the different intake times. Mean time variability was significantly lower in the morning than in the evening (34:16 min:s *vs*. 49:31 min:s; *p* = 0.05)

**Table 2:** Description of median intake time and time variability (t_VAR_) over three intake times for 78 patients. Parameters were calculated when at least three (median) or four (t_VAR_) records per intake time were available.

	Morning		Midday		Evening	
	Median [h:min]	t_VAR_ [min:s]	Median [h:min]	t_VAR_ [min:s]	Median [h:min]	t_VAR_ [min:s]
N	73	72	10	10	39	37
Mean	7:33	34:16	12:00	27:24	19:01	49:31
SD	1:00	28:50	00:33	29:37	1:35	50:43
Median	7:41	30:00	12:09	13:45	18:36	37:17
IQR	7:01–8:14	18:17–40:22	11:56–12:11	11:00–27:34	18:05–19:27	19:43–52:51
Range	4:00–9:23	00:43–228:45	10:28–12:35	6:51–103:26	16:02–23:26	02:43–250:34

Of 46 patients with more than one intake daily (Table 1), 38 had schedules that allowed for the calculation of intervals between morning and evening (see Table 3). Additional doses (midday and/or at night) were prescribed in 10 patients.

**Table 3:** Intervals between doses (mean ± SD) for 35 patients with morning-evening schedules (data of three patients were excluded from the calculation due to incomplete pairs).

Treatment schedule	N	Mean interval [h:min]
**Morning-Evening**	**35**	**11:38 ± 1:49**
**X-**0**-X-**0	25	11:48 ± 1:53
**X-**X**-X-**0	5	11:33 ± 1:01
**X-**0**-X-**X	2	10:10 ± 0:52
**X-**X**-X-**X	3	11:23 ± 3:07
**Morning-Midday**	**8**	**4:32 ± 1:04**
**Midday-Evening**	**7**	**6:33 ± 0:31**

### 3.3. Weekend-Effect

**Figure 1:**
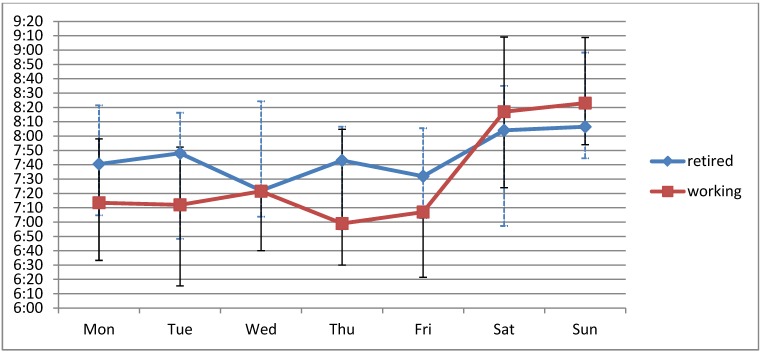
Median intake times of the morning doses in retired (N = 41) and working (N = 30) patients. Whiskers indicate the 1st and 3rd quartiles, respectively.

Mean intake times were significantly delayed on Saturday and Sunday compared to working days (*p* < 0.001). Consequently, the weekend days contributed significantly more to the overall drug intake variation than the working days (23.5 ± 12.7% *vs*. 10.6 ± 5.1%; *p* < 0.001). This effect was more pronounced in retired patients (N = 41; 30.0 ± 13.5%) than in working patients (N = 30; 18.4 ± 9.8%, *p* < 0.001) (Figure 1), but was independently observed in both groups. In absolute numbers, the mean t_VAR_ on working days was comparable in retired and working patients (22:48 ± 13:52 min:s *vs*. 22:23 ± 22:55 min:s, *p* = 0.92).

### 3.4. Socio-Demographic Factors

Time variability over the entire week differed significantly between retired and working patients (25:59 ± 13:44 min:s *vs*. 45:28 ± 39:28 min:s, *p* = 0.012) due to the weekend effect mentioned above. A tendency towards higher precision in timing adherence was observed in women compared to men (24:53 ± 13:44 min:s *vs*. 38:39 ± 33:08 min:s, *p* = 0.060), while no significant differences were found when patients were grouped by social status, smoking, prevention, treatment schedule (once daily *vs*. more than once daily) and MMAS-8 scores. Increased age correlated significantly with a more precise timing adherence (Spearman Rho = −0.382, *p* = 0.001). 

### 3.5. Treatment Scheme and Subjective Adherence

The number of concomitant drugs and the dosing frequency were not associated with time variability of drug intake. Patients’ beliefs and concerns, summarized by the BMQ differential score, were in good agreement with subjective adherence reported by the MMAS-8 score (R^2^ = 0.376, *p* = 0.001). This correlation was mainly driven by the BMQ concerns subscore, which significantly correlated with t_VAR_ (R^2^ = 0.242, *p* = 0.04).

### 3.6. Biomarker Response

Of the 68 patients with LLD, 22 (32.4%) did not reach their target LDL-C values and had a lower timing precision of the LLD intake compared to the 46 patients (67.6%) who reached their target LDL-C values (t_VAR_ = 67:44 ± 76:22 min:s *vs*. 28:05 ± 18:54 min:s, *p* = 0.011). A higher timing variation of the LLD intake correlated with higher LDL-C values (R^2^ = 0.323, *p* = 0.011). In parallel, patients with morning intake of the LLD had a tendency towards lower LDL-C values than patients with evening intake (2.3 ± 0.6mmol/L *vs*. 2.6 ± 0.7 mmol/L, *p* = 0.07), but this observation was confounded by a tendency towards higher potency of the LLDs in the morning group (Mann-Whitney U = 5.906, *p* = 0.05). The t_VAR_ of the LLD intake did not significantly differ between morning and evening LLD intakers (31:29 ± 19:36 min:s *vs*. 46:29 ± 59:03 min:s, *p* = 0.2). 

## 4. Discussion

### 4.1. Main Findings

Biomarker response is an intermediate outcome and can reflect the forgiveness of a drug. In HIV, asthma or blood pressure drugs, electronic adherence was predictive of biomarker outcomes [6,7,17]. Safety and effectiveness may be directly linked to the timing adherence to drugs with critical pharmacological properties. For the exemplary case of lipid lowering therapy, the impact of intake time variability can be estimated from its effects on LDL-cholesterol. LDL-C values typically change within a longer timeframe than in the short period of this study. However, significant time variability may occur, even in a short time frame. In this study, lower LDL-C values were achieved when a precise timing adherence with the LLD was observed, and those patients were more likely to reach their LDL-C targets. Remarkably, this finding was independent of the time of day, although advantages regarding the efficacy of LLD were attributed to the evening intake, at least for those agents with shorter elimination half-lives. Plakogiannis and Cohen found clinical evidence supporting the pharmacologically reasonable evening intake of simvastatin, while data for other statins remained inconclusive [18]. Statins are not known to be markedly sensitive regarding timing adherence. Nevertheless, the results presented here indicate that a regular timing of drug intake may be of more importance than the time of day for the optimization of statins’ effectiveness. Given the generally lower time variability in the morning intake times, and in light of the observed association between LDL-C values and the variation in drug intake t_VAR_, a morning intake of the LLD seems favorable. 

However, the limitations to a general recommendation for morning intake become evident when considering the remarkable differences of timing adherence pattern in specific patient groups. Retired patients were more likely to take their morning doses regularly over the entire week, while working patients showed a higher variability of the first daily dose due to a significantly delayed intake on Saturday and Sunday (weekend-effect). A plausible explanation for a lower t_VAR_ in the elderly, e.g. a higher valuation of drug therapy due to disease experiences, was not supported by BMQ scores, which were not age-dependent. Special care should be given to patients with higher concerns, since they showed a higher time variability of drug intake. The results presented here confirm previous reports on the ability of BMQ scores to predict subjective adherence as measured with the MMAS-8 [14]. 

No further contributors to high t_VAR_ could be identified in the sub-study. The use of a multidrug punch card may have facilitated the achievement of 100% taking adherence, especially for those patients with several intake times per day. Thus, occupational status remains the principal factor influencing electronically measured adherence. Further personalization of drug intake schedules should thus rely on the individual assessment of timing adherence collected by POEMS, unless future studies allow the prediction of timing adherence pattern from the patients’ socio-demographic and clinical characteristics.

When studying adherence to lipid lowering (LL) and antihypertensive (AH) drug therapy in a retrospective cohort of 8,506 patients using refill data and the proportion of days covered, Chapman *et al.* found the number of other prescriptions concomitant to LL and AH therapy to be the strongest predictor of non-adherence, followed by age, sex and the time between AH and LL therapy initiation [19]. In the presented study, neither the number of drugs nor the number of dosing times per day were associated with differences in objective measures of adherence, leading to the conclusion that the multidrug punch card reduced the complexity of the regimen to an irrelevant factor. Still, age, gender and occupational status remained important determinants of adherence.

### 4.2. Objectively Measured Adherence and Biomarkers

Balanced intervals between drug intakes are crucial to prevent fluctuations in plasma levels and to avoid the consequences of deprivation and subsequent onset of drug effect. Some authors emphasized the need to consider dosing intervals instead of the percentage of doses taken, which relies on a pharmacologically naive concept [9]. Time variability of drug intake should be interpreted in light of the duration of the action of a drug [10]. In the presented study, monitoring of patient’s multiple drug regimen was performed, and this enabled the comparison of timing adherence with the requirements of each drug. Unfortunately, forgiveness has not been characterized for every drug. Except for oral contraception, rationally based procedures to prevent the consequences of drug withdrawal are nonexistent. For drugs whose forgiveness exceeds the timing interval, efficacy should not be affected, but accumulation and toxicity might be more critical [20]. Considerations on time deviations from prescribed schedules have not yet led to regulatory consequences, thus only scarce data exist on time variability of drug therapy and clinical consequences in outpatients.

### 4.3. Strengths and Limitations

The strength of this study lies in the close monitoring of patient adherence with all oral solid drugs. One of the limitations is the use of unblinded electronic adherence monitoring, which is inherently associated with biased adherence [21]. Further, the limited duration of the monitored period and an artificial and highly adherence-enhancing short term setting may explain the extraordinary 100% adherence rate. Finally, the small sample size limits the impact and generalization of the results. However, data collected with similar methods are scarce and limit the possibility to put the presented findings in the context of previous research.

## 5. Conclusion

Collecting data on multidrug adherence with POEMS allowed the complete delineation of the patients’ pattern of timing adherence with all oral solid drugs. Variations in intake precision and in dose-to-dose intervals were measured, and with the proven 100% taking adherence over the observational period, they could be related to biomarker response. Overall, the intake time variability was more precise with morning intakes than with evening intakes, and a weekend effect contributed to a remarkable variability in working patients. In patients with lipid lowering therapy, a lower time variability of the LLD intake was associated with lower LDL-C values, independently of the time of day. Further research is needed to confirm the impact of timing adherence on the effectiveness of LLD. Future application of POEMS may provide data on adherence patterns and substantiate the rationale for personalized intake schedules based on individual adherence reports.
